# Cost-effectiveness analysis of malaria rapid diagnostic tests for appropriate treatment of malaria at the community level in Uganda

**DOI:** 10.1093/heapol/czw171

**Published:** 2017-02-15

**Authors:** Kristian S Hansen, Richard Ndyomugyenyi, Pascal Magnussen, Sham Lal, Siân E Clarke

**Affiliations:** 1 Department of Public Health, Section for Health Services Research, University of Copenhagen, Denmark; 2 Department of Global Health and Development, Faculty of Public Health and Policy, London School of Hygiene and Tropical Medicine, UK; 3 Vector Control Division, Ministry of Health, Kampala, Uganda; 4 Centre for Medical Parasitology and Microbiology & Institute of Veterinary Disease Biology, Faculty of Health and Medical Sciences, University of Copenhagen, Denmark; 5 Department of Disease Control, Faculty of Infectious and Tropical Diseases, London School of Hygiene and Tropical Medicine, UK

**Keywords:** Malaria, Community health workers, Community case management, Rapid diagnostic test, Artemisinin-based combination therapy, Cost-effectiveness analysis, Uganda

## Abstract

In Sub-Saharan Africa, malaria remains a major cause of morbidity and mortality among children under 5, due to lack of access to prompt and appropriate diagnosis and treatment. Many countries have scaled-up community health workers (CHWs) as a strategy towards improving access. The present study was a cost-effectiveness analysis of the introduction of malaria rapid diagnostic tests (mRDTs) performed by CHWs in two areas of moderate-to-high and low malaria transmission in rural Uganda. CHWs were trained to perform mRDTs and treat children with artemisinin-based combination therapy (ACT) in the intervention arm while CHWs offered treatment based on presumptive diagnosis in the control arm. Data on the proportion of children with fever ‘appropriately treated for malaria with ACT’ were captured from a randomised trial. Health sector costs included: training of CHWs, community sensitisation, supervision, allowances for CHWs and provision of mRDTs and ACTs. The opportunity costs of time utilised by CHWs were estimated based on self-reporting. Household costs of subsequent treatment-seeking at public health centres and private health providers were captured in a sample of households. mRDTs performed by CHWs was associated with large improvements in appropriate treatment of malaria in both transmission settings. This resulted in low incremental costs for the health sector at US$3.0 per appropriately treated child in the moderate-to-high transmission area. Higher incremental costs at US$13.3 were found in the low transmission area due to lower utilisation of CHW services and higher programme costs. Incremental costs from a societal perspective were marginally higher. The use of mRDTs by CHWs improved the targeting of ACTs to children with malaria and was likely to be considered a cost-effective intervention compared to a presumptive diagnosis in the moderate-to-high transmission area. In contrast to this, in the low transmission area with low attendance, RDT use by CHWs was not a low cost intervention.

**Key Messages**

- Involving community health workers (CHWs) is seen as a promising strategy towards improving access to prompt and appropriate diagnosis and treatment of malaria in Sub-Saharan Africa but cost-effectiveness information is limited.
- Cost-effectiveness analyses of introducing malaria rapid diagnostic tests (mRDTs) performed by CHWs on children under five and treatment with artemisinin-based combination therapy (ACT) was compared to a presumptive diagnosis in a moderate-to-high and a low malaria transmission area in rural Uganda.
- The introduction of mRDTs by CHWs resulted in a substantial improvement in appropriate treatment of malaria in both transmission settings.
- The incremental health sector cost of introducing mRDTs was estimated at US$3.0 per appropriately treated child under five (US$3.6 from a societal perspective) in the moderate-to-high transmission area and at US$13.3 (US$14.9 from a societal perspective) in the low transmission area.

## Introduction

Malaria remains a major cause of morbidity and mortality among children in sub-Saharan Africa, despite the presence of effective and low cost interventions including artemisinin-based combination therapy (ACT) and insecticide-treated bednets (Jones *et al.* 2003; Black *et al.* 2010; Liu *et al.* 2012; WHO 2015). An important reason for this failure to prevent malaria deaths is that children with fever do not access effective interventions or access them at a late stage (Jones *et al.* 2003; Darmstadt *et al.* 2005; Haines *et al.* 2007). Prompt diagnosis and appropriate treatment are crucial elements in reducing mortality, but can be hampered by the long distances families have to travel to reach the nearest public health facility and inadequate treatment services available at these facilities due to recurring stock-outs of antimalarials, staff shortages or a lack of diagnostic capacity for parasitological confirmation (Kamal-Yanni *et al.* 2012). Many malaria-endemic countries are characterised by a shortage of health workers and geographical inequities in access, with rural and hard-to-reach areas particularly underserved in terms of health services (WHO 2006). A thriving private sector partially fills this gap and common practice in Africa is to treat episodes of fever with over-the-counter medication purchased at drug retail outlets. Yet this may increase the risk of substandard treatment practices; e.g. sale of cheaper ineffective antimalarials rather than ACT, sale of partial doses, or sale of antimalarial drugs without parasitological confirmation (Whitty *et al.* 2008; Kamal-Yanni *et al.* 2012).

Against this background, increased deployment of community health workers (CHWs) has been seen as a promising strategy towards diminishing these problems (Haines *et al.* 2007; Young *et al.* 2012; Perry *et al.* 2014). While programs differ between countries, common features of CHWs are that they are usually volunteers recruited from their community; receive a short training; and work for little or no compensation (Ruizendaal *et al.* 2014). These characteristics have led to the expectation that CHWs can significantly expand access to key health services to underserved populations, deliver interventions promptly and at a lower cost than other health cadres in terms of salary and training.

Several studies have shown that CHWs are able to deliver prompt, appropriate malaria treatment in their communities (Sirima *et al.* 2003; Chinbuah *et al.* 2006; Staedke *et al.* 2009; Okwundu *et al.* 2013) and achieve cure rates above 90% among children with malaria parasites (Ajayi *et al.* 2008). The presence of CHWs can also result in reduced workload in public sector health facilities (Tiono *et al.* 2008; Lal *et al.* 2015). Following WHO guidelines that all suspected malaria cases should be parasitologically confirmed and treated with ACT if positive (WHO 2010), research has demonstrated CHWs’ capability to perform a malaria rapid diagnostic test (mRDT), interpret the result correctly and prescribe an appropriate treatment (Hawkes *et al.* 2009; Mubi *et al.* 2011; Chanda *et al.* 2011a; Counihan *et al.* 2012; Thiam *et al.* 2012; Ndyomugyenyi *et al.* 2016); although a range of factors can reduce success, including insufficient training (Harvey *et al.* 2008), accuracy of the mRDT utilised (Ruizendaal *et al.* 2014) and tendency to disregard negative mRDT results (Elmardi *et al.* 2009; Ruizendaal *et al.* 2014).

Less evidence is available on cost and cost-effectiveness of CHWs trained to diagnose and treat malaria, and studies vary considerably in the methodology chosen in terms of categories of costs included, effectiveness measure utilised and the diagnostic method of the CHWs (Vaughan *et al.* 2015). Using a Markov model and societal perspective cost data from Uganda, Lubell *et al.* (2010) concluded that presumptive diagnosis and treatment by CHWs would likely be cost-effective versus health facility care in high malaria transmission areas, but less likely in low transmission settings. Conversely, the incremental societal cost per disability-adjusted life year averted was found to be US$90.3 for an intervention where CHWs diagnosed children presumptively for malaria and treated with ACT relative to standard care at public health facilities and over-the-counter drugs in private outlets in Ghana (Nonvignon *et al.* 2012). Later studies, in which CHWs were trained to perform mRDTs and treat positives with ACT were also found to be more cost-effective than facility-based management in Zambia, where the provider cost per case correctly treated was US$4.2 and US$6.1 respectively (Chanda *et al.* 2011b). Cost per life saved for CHWs equipped with mRDTs and ACTs was slightly higher than management at public health facilities in Ethiopia (US$2258 versus US$2150) from a provider perspective (Gaumer *et al.* 2014). However, when analysed from a societal perspective, CHWs had a slightly lower cost per life saved than health facilities (US$884 versus US$922) due to household-level benefit in saved out-of-pocket spending. Compared to presumptive treatment, the potential economic benefits of mRDTs could include: cost-savings through reduced overuse of ACTs and improved patient outcomes resulting from targeting of ACTs to malaria cases, and detection of non-malarial fevers.

The present research presents a cost and cost-effectiveness analysis based on data from two cluster-randomised controlled trials to investigate the effect on appropriate malaria management of training CHWs to perform mRDT diagnosis in children and treat with ACT, in two areas with different levels of malaria transmission risk in rural Uganda (Ndyomugyenyi *et al.* 2016). We present the results of a comprehensive costing methodology including government provider cost, time cost of CHWs involved and costs incurred by households from initial CHW consultation and additional treatment seeking over the next 14 days.

## Methods

### Study area and population

The trials and cost data collection were conducted in Rukungiri District, South-Western Uganda, inhabited by the Bahororo and Bakiga ethnic groups, whose main occupation is subsistence farming. This district has a wide altitudinal range resulting in diverse malaria transmission patterns within the same district. Two sub-counties were selected as trial sites: Bwambara sub-county, a lower altitude area bordered by Lake Edward, which was meso-endemic for malaria with moderate-to-high transmission, and Nyakishenyi sub-county, a highland area with low transmission. All villages in the two sub-counties were invited to participate in the trial; a total of 63 villages in Bwambara and 64 villages in Nyakishenyi, ranging between 981–1203 and 1064–2157 metres above sea level in each sub-county respectively (Ndyomugyenyi *et al.* 2016).

## Study design

Cost-effectiveness analysis was conducted alongside two cluster-randomised controlled trials involving community-level provision of treatment for malaria in children under five by CHW volunteers. Details of the trials are described elsewhere (Ndyomugyenyi *et al.* 2016; www.actconsortium.org/RDThomemanagement). In brief: the primary trial objective was to evaluate the impact of mRDTs used by CHWs (intervention) compared to presumptive clinical diagnosis by CHWs (control) on the proportion of patients receiving appropriately-targeted ACT treatment; all villages in each sub-county were invited to participate; a public meeting was held in each village to explain the purpose of the research and community members asked to select three CHWs per village. Using village as the unit of randomisation, villages within each transmission area were randomly allocated to either the intervention or control arm and CHWs in a village all belonged to the same study arm.

CHWs in both study arms were trained to recognise signs and symptoms of malaria, how to administer antimalarial treatment to children under five, and when to refer. Half the villages (intervention villages) were randomised to receive an additional one-day training in malaria diagnosis using mRDTs and to give an ACT only after a positive test result. CHWs were trained to treat mRDT-positive children with danger signs with rectal artesunate pre-referral treatment, and refer these children to the nearest public health facility. CHWs were also trained to refer, irrespective of the test result, if the child had any danger signs or other specific listed symptoms. Artemether-lumefantrine (AL) and mRDTs were provided at no cost to participating CHWs, and CHW services were free to community members. CHWs in the control arm offered identical services, except that diagnosis of malaria was based on clinical signs and symptoms only, as was the decision to refer children.

Additional supporting interventions included community sensitisation to convey key messages such as: not all fevers are malaria and hence a diagnostic test was advisable before treatment with ACT, and that mRDTs were available at government health facilities and from some CHWs. For the first four months after training, close support supervision visits were provided regularly by the research team to assist CHWs with problems or questions related to their services. Supervision was scaled back after this initial period. No formal remuneration was given to CHWs for their services, but CHWs did receive a monthly soap and paraffin allowance, and bicycles to facilitate their collection of supplies and to attend supervision meetings.

### Estimation of effect

Measurement of effect was determined from the cluster-randomised trials (Ndyomugyenyi *et al.* 2016). Data were collected on all children under five who presented with fever to a CHW during the trial period. Treatment decisions made by CHWs were evaluated against the results of later microscopy of a reference blood slide collected at each consultation. Slides were examined twice by two experienced laboratory technicians and discrepant results were resolved by a third technician blinded to previous results. The measure of effect was ‘appropriate treatment of malaria with ACT’, which was a composite indicator defined as: a child under five with malaria parasites in their blood (confirmed by expert microscopy) who received a course of ACT or rectal artesunate, or a child with a negative microscopy result who was not prescribed an artemisinin-based treatment. This indicator was developed to be able to measure the targeting of ACT exclusively to those patients with malaria parasites in their blood. Previous research studies have utilised this indicator to assess the impact of introducing mRDTs on appropriate malaria treatment including in public health centres in two areas of Ghana (Ansah *et al.* 2013; Baiden *et al.* 2016), in private drug shops in Uganda (Mbonye *et al.* 2015) and in public health centres in Afghanistan (Leslie *et al.* 2014). For the present research, the proportion of children treated appropriately by CHWs in each study arm, within each transmission setting, was measured over a 12-month period from January-December 2011.

### Estimation of costs

The value of resources utilised in each study arm was measured from both public health sector and societal perspectives and presented separately. Costs measured from the societal perspective incorporated the value of time committed by the CHWs, costs borne by households and health sector costs. All cost figures were adjusted to the 2011 price level using an annual inflation rate of 8.8% corresponding to the average annual increase in the GDP deflator in Uganda from 2004 to 2013 and presented in US dollars (UGX2523 = US$1) (World Bank 2015).

### Health sector costs

Total costs of resources for training of CHWs, supervision, and community sensitisation were considered a health sector cost since the government would need to fund these activities if the intervention was to be implemented at scale. Personnel costs of these activities were obtained through interviews with staff who were asked about their gross monthly salary and to estimate the number of days spent on these activities. All other costs were captured from the financial accounting system of the research project. Training of CHWs, including the initial period of close support supervision, and community sensitisation can be expected to have a useful lifespan beyond the evaluation period of a single calendar year and were treated as capital goods with annual equivalents calculated assuming a lifespan of 5 years and a real discount rate of 3% (Walker and Kumaranayake 2002). The less intense routine supervision conducted during the 12-month evaluation period was considered a recurrent cost and measured for the 2011 calendar year.

mRDTs, drugs and other consumables were supplied free to CHWs and the cost of acquiring these commodities was also considered a health sector cost. The mRDT used, First Response®, was available in Uganda in 2011 at US$0.87 per test, including shipping. Adding an assumed wastage rate of 5%, and costs of sterile gloves, cotton wool, and spirit additionally needed to perform the test, resulted in a unit cost of US$1.00 per mRDT performed. The median price per course of ACT treatment with AL as well as rectal artesunate was obtained from an international drug price list (Management Sciences for Health 2012). A recommended ten percent was added for shipping (Management Sciences for Health 2012) and another 12.5% to cover storage and quality control in-country (personal communication, Central Medical Stores). Assuming 10% wastage, the estimated cost per course of AL in 2011 was US$0.77 for a child below 3 years of age and US$1.63 for a child aged 3–7 years, and US$0.74 for a treatment with rectal artesunate. Bicycles and petroleum lamps supplied to CHWs were valued at prevailing local prices in Uganda.

Since CHWs were trained to refer children with danger signs to the nearest public health facility, costings were carried out for two public health centres in each transmission setting with the aim of estimating the treatment cost per health facility visit for referred children. Recurrent expenditure for each health centre for the financial year 2011/12 was obtained from the Rukungiri District Office. Capital expenditure was estimated as the annualised value (Walker and Kumaranayake 2002) of the construction cost of health centre buildings and 2011 purchase prices of the main equipment and furniture found at the four health centres visited. A discount rate of 0.03 and expected life spans of 7, 10 and 30 years were used for equipment, furniture and buildings respectively. Following the standard step-down costing method (Conteh and Walker 2004; Drummond *et al.* 2005), aggregate health centre level costs for 2011/12 were allocated in a step-wise fashion to overhead services (i.e. administration, cleaning) and intermediate services (dispensary, diagnostics) and finally to patient services (direct patient care). The step-down costing was supplemented by micro-costing methods (Brouwer *et al.* 2001) in order to separate out the cost of diagnosis by mRDT and treatment of malaria and non-malaria fevers in the outpatient departments of the health centres. The cost of the recommended drugs and doses for treatment of a child under five were estimated using an international price list (Management Sciences for Health 2012), assuming that health workers generally followed official clinical guidelines (Ministry of Health 2010). Relevant personnel were interviewed to estimate time required per outpatient treated, which was then valued according to salary level of the personnel. Combining the data collected through the standard step-down and micro costing methods resulted in the unit cost per visit for the treatment of malaria and non-malarial fever which incorporated both cost of direct resource input (e.g. drugs and personnel time) as well as the cost of overhead activities and support activities from four public health facilities.

### Costs borne by community health workers

CHWs invested time in the programme without receiving any formal monetary remuneration. The opportunity cost of their time was valued at US$1.21 per day corresponding to the GDP per capita per day in 2011 (World Bank 2015); a value that was assessed as part of the sensitivity analysis. The rationale behind this choice was the human capital approach according to which the value of time of an individual is equal to what he/she contributes to society in the form of the market value of output produced by the individual in his/her normal work (Shillcutt *et al.* 2009). This approach was modified for this research by setting the value of time as equal for all individuals (irrespective of their normal work) at the level of GDP per capita – the average contribution of Ugandans to output. Time utilised to diagnose and treat a child and for overhead activities such as travel and time spent attending supervisory meetings and collecting supplies was captured from interviews with a subset of CHWs.

### Household costs

Household costs in families with a sick child were captured for a two-week period starting from the initial visit at a CHW. Trained interviewers visited families two weeks after CHW treatment and interviewed the main caregiver of the child to capture information on all out-of-pocket expenditure in this period, including: costs incurred at the time of initial CHW visit, completed referrals to public health facilities, any additional treatment-seeking at private sector providers, and special foods purchased for the sick child; as well as time utilised for treatment-seeking and days the main caregiver was unable to perform their normal activities due to caring for the child. Lost time was valued at US$1.21 per day. Household costs were measured in a sample of 413 families: 293 households in the moderate transmission setting (133 and 160 in the mRDT and presumptive arms respectively) and 120 in the low transmission setting (47 and 53 in each arm). Sample size for household cost interviews was calculated to test a hypothesis that out-of-pocket expenditure would be lower in the mRDT arm. To detect a decrease of at least 30% in mean out-of-pocket expenditure in the intervention arm from a mean cost of UGX3500 in the control arm, in a trial with 10 clusters per arm and assuming k = 0.25, power of 80%, and significance level of 5%, required 250 interviews per arm (Hayes and Bennett 1999).

### Cost-effectiveness analysis

**Figure 1. (a):**
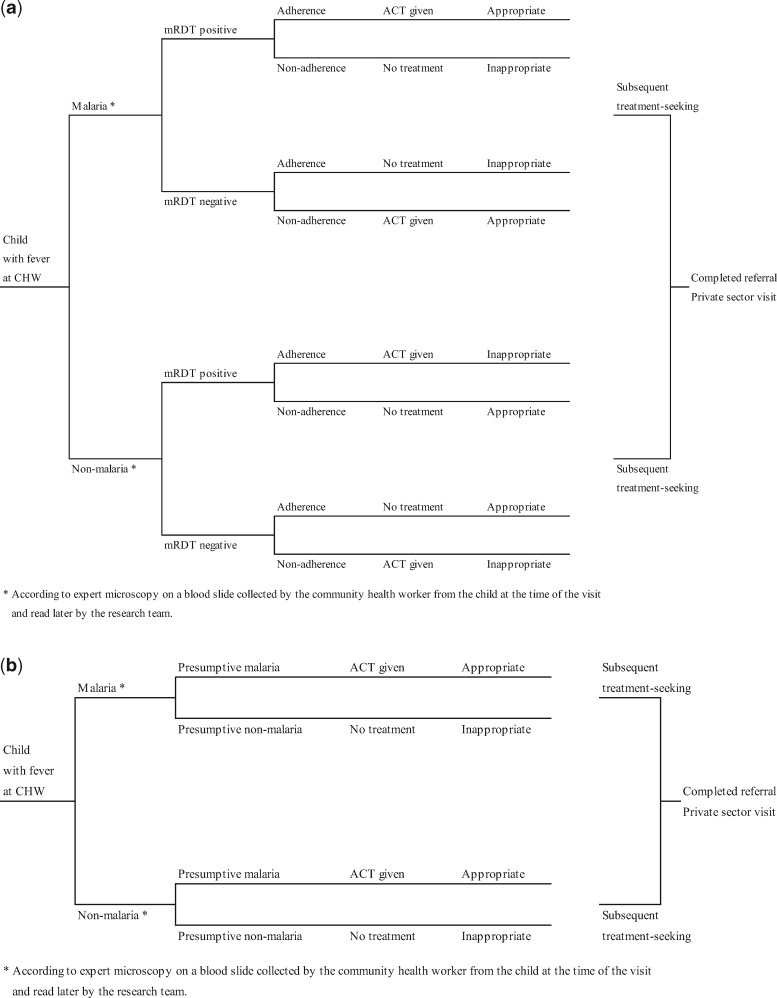
Decision model for children under five visiting community health workers offering malaria diagnosis by rapid diagnostic test and **(b)** Decision model for children under five visiting community health workers offering presumptive malaria diagnosis.

Data on the costs and effects were linked through a decision analytical approach (Briggs *et al.* 2006) using the decision trees displayed in Figure 1a and b. Identical decision tree structures were used in the two transmission settings. Probabilities of individual decision tree branches indicating malaria status (reference microscopy), mRDT accuracy (sensitivity and specificity), presumptive diagnosis, adherence to mRDT result and decision by households to follow referral advice were derived using the data available from the trial (Ndyomugyenyi *et al.* 2016). The probability of additional treatment-seeking in the private sector was captured from the sub-sample of caregivers interviewed to capture household costs. At the end of each decision tree, treatment of a child with fever was classified as appropriate or not according to the study definition used for the primary trial outcome. Decision tree probabilities differed by the arm and transmission setting and have been listed in Supplementary File 1a and 1b.

Health sector and societal costs by study arm in each transmission setting were calculated by populating the decision trees with cost per CHW visit estimates as described above. Annualised costs estimated for community sensitisation, training, supervision, allowances and supplies for CHWs were divided by the number of visits to CHWs by the arm and setting in the 12-month period January-December 2011. These average cost per visit estimates therefore depended on the number of children seen in a particular arm and setting. Cost per referral visit to public health centres for treatment of malaria and non-malarial fevers entered the decision model as an average of the unit costs obtained from the four health centres included in facility costing. Average household out-of-pocket expenditure per event and the opportunity cost of time lost per illness episode were obtained from the 413 household cost interviews. All costs per event estimates utilised in the decision model are listed in Supplementary File 1a and 1b.

Total health sector and societal costs and number of appropriately-treated children were calculated by letting 1000 children pass through the populated decision tree for each arm and transmission setting. The incremental cost-effectiveness ratio (ICER) was obtained by subtracting total costs in the control arm from the total costs in the intervention arm (numerator) and subtracting the total effect measured as appropriately treated children in the control arm from the total effect in the intervention arm (denominator). The ICER for each transmission setting therefore measured the extra cost per additional appropriately treated child when CHWs are given mRDTs for diagnosis instead of making a presumptive diagnosis based solely on signs and symptoms.

### Sensitivity analysis

One-way sensitivity analyses were performed by changing the values of individual parameters and assumptions in the decision models to observe the effect on the ICER. The parameters assessed included malaria prevalence among patients seen; accuracy of mRDT; adherence to test results; prices of mRDTs and ACTs; the opportunity cost of lost time; and cost per visit to public health centres. In addition, since CHWs in the low transmission area saw substantially fewer patients than CHWs in the moderate-to-high transmission setting, a sensitivity analysis was performed assuming that one CHW per village (rather than three) would be sufficient to meet service demand in communities with low malaria transmission.

Probabilistic sensitivity analysis (PSA) was undertaken to assess the sensitivity of the ICER to simultaneous variation in relevant parameters (Briggs *et al.* 2006) by defining probability distributions to selected decision model parameters rather than point estimates. Beta distributions were used for all the branch probabilities used in the decision trees using trial data to estimate distribution parameters (Ndyomugyenyi *et al.* 2016). Due to the non-negative, right-skewed nature of the household cost data, gamma distributions were used to describe the variation in these costs (Briggs *et al.* 2006) with distribution parameters obtained from the household cost survey interviews. Other cost parameters (e.g. health centre cost per visit) entered the analysis as point estimates as there were insufficient data available to derive the distributions. All distributions and point estimates used in the PSA are shown in Supplementary Material, Table S1a and b. Simultaneous selection of values from these parameter distributions and point estimates, followed by calculation of ICERs, was performed 10 000 times in Excel (Microsoft, Seattle) in order to propagate uncertainty in the ICERs (Doubilet *et al.* 1984). Uncertainty surrounding the ICERs was summarised by plotting joint incremental costs and incremental effects in the cost-effectiveness plane, and quantified by calculating confidence intervals and cost-effectiveness acceptability curves (CEACs) (Löthgren and Zetraeus 2000; O’Brien and Briggs 2002; Fenwick *et al.* 2004) which show the probability that use of mRDTs to inform the management of malaria by CHWs is cost-effective for different levels of a health policy maker’s hypothetical willingness-to-pay (WTP) for an appropriately treated child under five.

### Ethical approval

This study was approved by the Uganda National Council for Science and Technology and the London School of Hygiene & Tropical Medicine Ethics Committee. Patients refusing an mRDT test received presumptive treatment. The study was registered with ClinicalTrials.gov. Identifier NCT01048801 on 13th January 2010.

## Results

Cost and effects are presented for a standard population of 1000 children under five visiting CHWs by study arm for each transmission setting. In the moderate-to-high transmission area, use of mRDTs to diagnose malaria rather than by symptoms alone resulted in a substantial increase in the number of appropriately treated children of 485 per 1000 (95% CI 453 to 516) (Table 1a). The extra cost from a health sector perspective in the mRDT arm compared to the presumptive arm was US$1,462 per 1000 children (95% CI US$1,424 to US$1,500) leading to an ICER of US$3.0 (95% CI US$2.8 to US$3.3). In other words, use of mRDT diagnosis in contrast to presumptive diagnosis would cost the health sector US$3.0 per additional appropriately treated child under five. Applying a broader societal perspective incorporating health sector and household costs, the extra cost in the mRDT arm compared to the presumptive arm was US$1,755 per 1000 children (95% CI -US$7,740 to US$10 705) leading to an ICER of US$3.6 (95% CI -US$15.8 to US$22.5). In the low transmission setting (Table 1b), the performance of mRDT diagnosis resulted in an even greater increase in the number of appropriately treated children of 822 per 1000 (95% CI 785 to 848) compared to presumptive diagnosis. This improvement was mainly due to the high proportion of children not suffering from malaria that were not prescribed an ACT. Extra health sector cost in the mRDT arm compared to the presumptive arm was US$10 924 per 1000 children (95% CI US$10 878 to US$10 973) leading to an ICER of US$13.3 (95% CI US$12.9 to US$13.8). In the low transmission setting, diagnosing malaria using mRDTs instead of presumptive diagnosis would therefore cost the health sector US$13.3 per additional appropriately treated child under five. The extra societal cost in the mRDT arm compared to the presumptive arm was US$12 283 per 1000 children (95% CI US$2,783 to US$21 759) leading to an ICER of US$14.9 (95% CI US$3.4 to US$26.4).

**Table 1a.** Costs and effects in a standard population of 1000 children suspected of malaria by study arm and incremental cost-effectiveness ratio (ICER) of replacing presumptive diagnosis by rapid diagnostic tests performed by community health workers in a moderate-to-high transmission area in Rukungiri District, Uganda, 2011 (US$1 = UGX2,523)

	—— mRDT arm ——		– Presumptive arm –	
	***N***	***%***	***N***	***%***
**Children suspected of malaria**	**1,000**	**100**	**1,000**	**100**
Malaria (according to reference diagnosis)	378	38	302	30
Treated with ACT	375	38	994	99
Appropriately treated^a^	793	79	308	31
	**US$**	**%**	**US$**	**%**
**Health sector cost per 1000 children**	**5,674**	**65**	**4,213**	**60**
Community sensitisation	143	2	112	2
Training^b^	408	5	273	4
Supervision	1,227	14	697	10
Allowances for CHWs	1,609	18	1,270	18
Equipment for CHWs	363	4	286	4
mRDTs supplied	933	11	0	0
ACTs prescribed	489	6	1,208	17
Other supplies	396	5	366	5
Completed referrals to public health centres	106	1	1	0
**CHW cost (value of time) per 1000 children**	**244**	**3**	**185**	**3**
Overhead activities^c^	119	1	90	1
Diagnosis and treatment	125	1	96	1
**Household cost per 1000 children**	**2,857**	**33**	**2,623**	**37**
CHW visit (initial visit)	36	0	29	0
Completed referrals to public health centres	28	0	0	0
Drugs, fees, travel (private sector visits)	301	3	172	2
Special food to improve health	926	11	1,157	16
Opportunity cost of time lost	1,566	18	1,264	18
**Total societal costs per 1000 children**	**8,776**	**100**	**7,021**	**100**
**Incremental analysis**				
**(Replace presumptive diagnosis by mRDT diagnosis**				
**in 1000 children suspected of malaria)**				
Incremental number of appropriately treated [95% CI]		485	[453; 516]	
Incremental health sector cost, US$[95% CI]		1,462	[1,424; 1,500]	
Incremental societal cost, US$[95% CI]		1,755	[-7,740; 10,709]	
ICER health sector perspective, US$[95% CI]		3.0	[2.8; 3.3]	
ICER societal perspective, US$[95% CI]		3.6	[-15.8; 22.5]	

a Child with a positive reference diagnosis prescribed an ACT or child with a negative reference diagnosis not prescribed an ACT.

b Including cost of initial period of close support supervision.

c Quarterly review meetings, collection of supplies, communication with supervisors, etc.

In the moderate-to-high transmission setting, the health sector bore over 60% of the total societal cost (in both arms) with supervision and allowances for CHWs constituting the most important cost drivers. The cost of consumables (mRDTs, ACTs and other supplies) amounted to 22% of the total societal cost in both arms, with the additional costs of mRDTs being largely offset by savings in the cost of ACTs in the intervention arm. Use of mRDTs was associated with an increased probability of referral and seeking additional care at public health centres (4.5% in the mRDT arm and <0.1% in the presumptive arm), resulting in increased costs for both the public health sector and households in the mRDT arm. The probability of households seeking further care at private sector providers was also higher in the mRDT arm: 8.7% vs. 5.0% in the presumptive arm, comprising 3% and 2% of societal cost respectively. Overall, approximately one third of the total societal cost was borne by households with an opportunity cost of self-reported time required to look after an ill child as the most important component. The opportunity cost of CHWs’ time contributed only 3% to the total societal cost in both arms.

The health sector cost in the low transmission setting constituted a much larger share of total societal cost at 82% and 77% in the mRDT and presumptive arms respectively. The distribution of costs was similar to the moderate-to-high transmission setting, with supervision and allowances for CHWs being the largest cost component. The probability of seeking additional care at public health centres was 8.7% and 1.8% in the mRDT and presumptive arms respectively, resulting in < 1% of the total societal cost in both arms. The probability of seeking further care at private suppliers like drug shops and the local mission hospital was 35.7% and 11.4% in the mRDT and presumptive arms, resulting in 5% and 3% of societal cost respectively. The opportunity cost of CHWs’ time comprised 2% of the total societal cost in both arms. Although overall household cost constituted a lower share of societal cost in the low transmission area (16-21%), the opportunity cost of self-reported time spent caring for a sick child was again the most important component.

Levels of cost varied across settings and arms. Absolute health sector cost in the low transmission area was much higher than in the moderate-to-high transmission setting (both arms) (Tables 1a and 1b). Similarly, health sector cost in mRDT arms in both transmission settings was higher than the presumptive arms. This was a reflection of differences in consultation rates observed during the 2011 evaluation period (Ndyomugyenyi *et al.* 2016) where more children sought care in the moderate-to-high transmission area compared to the low transmission site, and fewer children visited CHWs with mRDTs than CHWs offering presumptive treatment (in both settings). Some health sector activities, including community sensitisation, training, supervision and allowances and equipment for CHWs are determined primarily by the number of treatment providers, rather than the number of patients. The average cost per child was as a consequence higher in study arms with fewer children seen in the evaluation period thus explaining some of the differences in health sector cost by arm and setting.

**Table 1b.** Costs and effects in a standard population of 1000 children suspected of malaria by study arm and incremental cost-effectiveness ratio (ICER) of replacing presumptive diagnosis by rapid diagnostic tests performed by community health workers in a low transmission area in Rukungiri District, Uganda, 2011 (US$1 = UGX2,523)

	—— mRDT arm ——		– Presumptive arm –	
	***N***	***%***	***N***	***%***
**Children suspected of malaria**	**1,000**	**100**	**1,000**	**100**
Malaria (according to reference diagnosis)	60	6	56	6
Treated with ACT	69	7	968	97
Appropriately treated^a^	901	90	78	8
	**US$**	***%***	**US$**	***%***
**Health sector cost per 1000 children**	**22,971**	**82**	**12,047**	**77**
Community sensitisation	899	3	462	3
Training^b^	2,579	9	1,141	7
Supervision	4,453	16	1,788	11
Allowances for CHWs	10,349	37	5,546	35
Equipment for CHWs	2,334	8	1,251	8
mRDTs supplied	933	3	0	0
ACTs prescribed	72	0	1,078	7
Other supplies	1,177	4	748	5
Completed referrals to public health centres	175	1	33	0
**CHW cost (value of time) per 1000 children**	**579**	**2**	**333**	**2**
Overhead activities^c^	454	2	230	1
Diagnosis and treatment	125	0	103	1
**Household cost per 1000 children**	**4,374**	**16**	**3,262**	**21**
CHW visit (initial visit)	164	1	14	0
Completed referrals to public health centres	49	0	9	0
Drugs, fees, travel (private sector visits)	1,371	5	439	3
Special food to improve health	960	3	1,200	8
Opportunity cost of time lost	1,829	7	1,600	10
**Total societal costs per 1000 children**	**27,925**	**100**	**15,642**	**100**
**Incremental analysis**				
**(Replace presumptive diagnosis by mRDT diagnosis**				
**in 1000 children suspected of malaria)**				
Incremental number of appropriately treated [95% CI]		822	[795; 848]	
Incremental health sector cost, US$[95% CI]		10,924	[10,878; 10,973]	
Incremental societal cost, US$[95% CI]		12,283	[2,783; 21,759]	
ICER health sector perspective, US$[95% CI]		13.3	[12.9; 13.8]	
ICER societal perspective, US$[95% CI]		14.9	[3.4; 26.4]	

a Child with a positive reference diagnosis prescribed an ACT or child with a negative reference diagnosis not prescribed an ACT.

b Including cost of initial period of close support supervision.

c Quarterly review meetings, collection of supplies, communication with supervisors, etc.

One-way sensitivity analyses for the moderate-to-high transmission setting showed that the ICERs from both the health sector and the societal perspectives were robust to changes in most parameters investigated with a few important exceptions (Table 2a). Increasing malaria prevalence among children with fever to 50% rather than the 30-38% observed, resulted in an ICER which was two-fold higher than the central estimate making mRDT diagnosis by CHWs less attractive from a cost-effectiveness perspective. Decreasing adherence to negative mRDT results from the observed 98% to 70% or below had a large upward effect on the ICERs, making mRDTs less cost-effective. The number of children visiting CHWs was an important factor influencing the ICER levels. For instance, a 40% increase in consultations at CHWs with mRDTs, substantially reduced the ICER from a health sector perspective to US$0.7; and would make mRDTs more cost-effective relative to presumptive diagnosis.

**Table 2a.** Sensitivity to selected parameters of the incremental cost-effectiveness ratio (ICER) of replacing presumptive diagnosis by rapid diagnostic tests performed by community health workers in moderate-to-high transmission area, Rukungiri District, Uganda, 2011 (US$1 = UGX2523)

	—- ICER in US$—-				—- ICER in US$—-	
	Health				Health	
Parameter^a^	sector	Societal		Parameter^a^	sector	Societal
Malaria prevalence among				Health centre cost for		
customers (38% and 30%)				treatment and diagnosis		
10%	1.8	2.2		50% lower	2.9	3.5
20%	2.2	2.7		30% lower	2.9	3.6
40%	3.8	4.6		30% higher	3.1	3.7
50%	5.6	6.7		50% higher	3.1	3.7
Sensitivity of mRDT (73%)				Probability of subsequent		
60%	3.2	3.9		treatment-seeking in		
80%	2.9	3.5		mRDT arm (9%)		
90%	2.8	3.3		5%	3.0	3.3
100%	2.7	3.2		20%	3.0	4.4
Specificity of mRDT (83%)				30%	3.0	5.2
60%	4.7	5.5		Probability of subsequent		
70%	3.8	4.5		treatment-seeking in		
90%	2.7	3.3		presumptive arm (5%)		
100%	2.3	2.8		10%	3.0	3.3
Adherence to negative				20%	3.0	2.5
mRDT (99%)				30%	3.0	1.8
60%	5.5	6.5		ACT price		
70%	4.7	5.5		20% lower	3.3	3.9
80%	4.0	4.7		40% lower	3.6	4.2
Change in number of				50% lower	3.7	4.3
children visiting				mRDT price (US$0.70)		
CHWs in mRDT arm				20% lower	2.6	3.2
40% lower	8.4	9.1		40% lower	1.8	2.4
20% lower	5.0	5.7		50% lower	2.0	2.6
20% higher	1.7	2.2		Discount rate (3%)		
40% higher	0.7	1.3		1%	3.0	3.6
Change in number of				7%	3.1	3.7
children visiting				10%	3.2	3.8
CHWs in presumptive arm				Community sensitisation,		
40% lower	−0.8	−0.3		training and intense		
20% lower	1.6	2.2		supervision (every 5 years)		
20% higher	4.0	4.6		Every 2 years	3.5	4.1
40% higher	4.6	5.3		Every 7 years	2.9	3.5
Probability of completed				Opportunity cost		
referral if negative				per day (US$1.2)		
diagnosis (8% and 0%)				US$0.2	3.0	3.0
20%	3.3	4.1		US$0.6	3.0	3.2
40%	3.9	4.8		US$1.6	3.0	3.9
50%	4.1	5.2		US$2.0	3.0	4.1

a Actual parameter value observed in the trial (Ndyomugyenyi et al. 2016) is shown in parenthesis.

One-way sensitivity analyses for the low transmission area also found that the ICERs from both the health sector and the societal perspectives were robust to changes in most of the parameters investigated (Table 2b). Malaria prevalence, adherence by CHWs to mRDT results and attendance of children at CHWs again constituted the only exceptions. Given the low attendance rate in the low transmission area, it is possible that one CHW rather than three would be sufficient to handle the current demand which could lead to cost savings with respect to training, supervision and allowances and equipment for CHWs (Supplementary Material, Table S2). Overall, this additional sensitivity analysis indicated a much lower ICER from a health sector perspective of US$5.4 (95% CI US$5.2 to US$5.7), and US$6.9 (95% CI -US$4.4 to US$18.0) from a societal perspective compared to the central estimates presented in Table 1b.

**Table 2b.** Sensitivity to selected parameters of the incremental cost-effectiveness ratio (ICER) of replacing presumptive diagnosis by rapid diagnostic tests performed by community health workers in low transmission area, Rukungiri District, Uganda, 2011 (US$1 = UGX2523)

	—- ICER in US$—-			—- ICER in US$—-	
	Health			Health	
Parameter^a^	sector	Societal	Parameter^a^	sector	Societal
Malaria prevalence among			Health centre cost for		
customers (6% and 6%)			treatment and diagnosis		
15%	16.2	18.2	50% lower	13.2	14.8
20%	18.3	20.6	30% lower	13.2	14.9
25%	21.1	23.7	30% higher	13.3	15.0
30%	24.9	28.0	50% higher	13.4	15.0
Sensitivity of mRDT (21%)			Probability of subsequent		
40%	13.1	14.8	treatment-seeking in		
60%	13.0	14.6	mRDT arm (36%)		
80%	12.8	14.4	5%	13.3	13.4
90%	12.7	14.3	20%	13.3	14.2
Specificity of mRDT (98%)			50%	13.3	15.6
60%	17.5	19.7	Probability of subsequent		
70%	16.2	18.2	treatment-seeking in		
80%	15.0	16.9	presumptive arm (11%)		
90%	14.0	15.8	2%	13.3	15.4
Adherence to negative			20%	13.3	14.5
mRDT (95%)			30%	13.3	14.0
60%	21.9	24.6	ACT price		
70%	18.6	20.8	20% lower	13.5	15.2
80%	16.1	18.0	40% lower	13.8	15.4
Change in number of			50% lower	13.9	15.5
children visiting			mRDT price (US$0.70)		
CHWs in mRDT arm			20% lower	13.0	14.7
40% lower	30.7	32.8	40% lower	12.6	14.2
20% lower	19.8	21.6	50% lower	12.7	14.3
20% higher	8.9	10.5	Discount rate (3%)		
40% higher	5.8	7.3	1%	13.0	14.7
Change in number of			7%	13.8	15.5
children visiting			10%	14.2	15.9
CHWs in presumptive arm			Community sensitisation,		
40% lower	4.6	6.1	training and intense		
20% lower	10.0	11.6	supervision (every 5 years)		
20% higher	15.4	17.1	Every 2 years	16.5	18.1
40% higher	17.0	18.7	Every 7 years	12.7	14.3
Probability of completed			Opportunity cost		
referral if negative			per day (US$1.2)		
diagnosis (9% and 24%)			US$0.2	13.3	14.5
20%	13.5	15.3	US$0.6	13.3	14.6
40%	14.0	15.9	US$1.6	13.3	15.1
50%	14.2	16.2	US$2.0	13.3	15.3

a Actual parameter value observed in the trial (Ndyomugyenyi et al. 2016) is shown in parenthesis.

**Figure 2.**
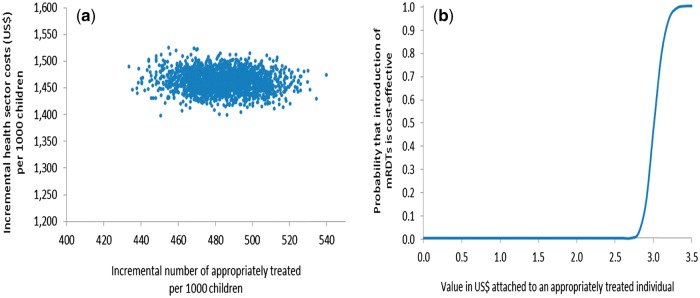
Probabilistic sensitivity analysis from a health sector perspective, moderate-to-high transmission area, Rukungiri District: (a) scatter plot of incremental health sector cost in US$ and incremental number of appropriately treated children resulting from replacing clinical diagnosis of malaria by rapid diagnostic test, 2011 (US$1 = UGX2,523) and (b) cost-effectiveness acceptability curve.

**Figure 3.**
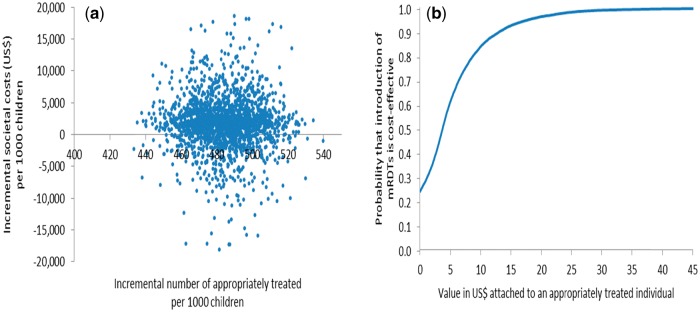
Probabilistic sensitivity analysis from a societal perspective, moderate-to-high transmission area, Rukungiri District: (a) scatter plot of incremental societal cost in US$ and incremental number of appropriately treated children resulting from replacing clinical diagnosis of malaria by rapid diagnostic test, 2011 (US$1 = UGX2,523) and (b) cost-effectiveness acceptability curve.

**Figure 4.**
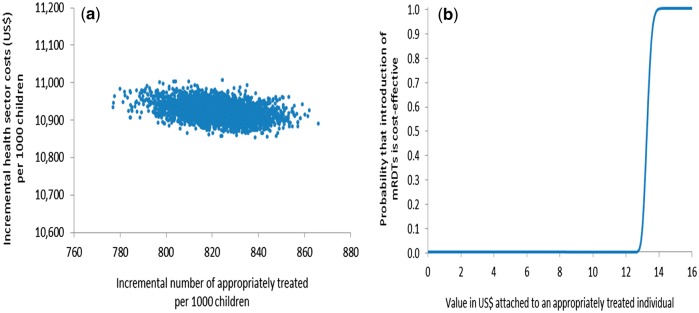
Probabilistic sensitivity analysis from a health sector perspective, low transmission area, Rukungiri District: (a) scatter plot of incremental health sector cost in US$ and incremental number of appropriately treated children resulting from replacing clinical diagnosis of malaria by rapid diagnostic test, 2011 (US$1 = UGX2,523) and (b) cost-effectiveness acceptability curve.

**Figure 5.**
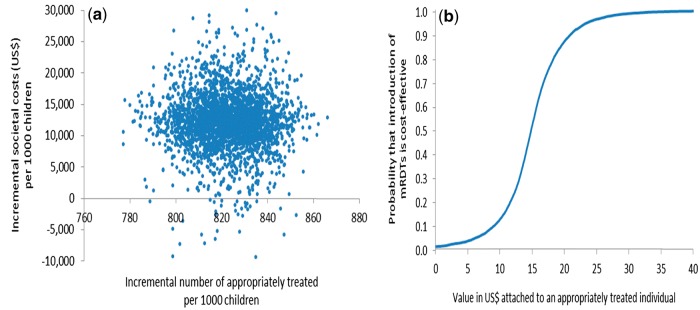
Probabilistic sensitivity analysis from a societal perspective, low transmission area, Rukungiri District: (a) scatter plot of incremental societal cost in US$ and incremental number of appropriately treated children resulting from replacing clinical diagnosis of malaria by rapid diagnostic test, 2011 (US$1 = UGX2,523) and (b) cost-effectiveness acceptability curve.

Results from the PSAs from both transmission settings are presented in Figures 2–5. In the moderate-to-high transmission area, all pairs of incremental health sector costs and effects were situated in the north-eastern quadrant of the cost-effectiveness plane meaning that the change in number of appropriately treated children was always positive and incremental health sector cost always positive if mRDTs were used (Figure 2a). According to the CEAC derived from this pattern of pairs of incremental health sector cost and effects (Figure 2b), the probability of mRDTs being a cost-effective intervention in a community-treatment programme was 46% if a health policy maker was willing-to-pay (WTP) at least US$3.0 per appropriately treated child, increasing to 94% and 100% if WTP was US$3.2 and US$3.5. This indicates a high probability that mRDTs would be cost-effective from a health sector perspective even at very low WTP in the moderate-to-high transmission setting. The PSA from a societal perspective showed greater uncertainty with respect to mRDT use as seen by the larger spread of incremental societal cost and effects (Figure 3a). From a societal perspective, much higher WTP would be required to deem use of mRDTs to be a cost-effective intervention (Figure 3b). Assuming that the WTP was US$5, the probability of mRDT use being cost-effective in the moderate-to-high transmission area was 61%; increasing to 84% and 93% if the WTP was US$10 and US$15 respectively.

The PSA conducted for the low transmission area showed a similar pattern (Figures 4 and 5), though the levels of incremental cost and effects were generally higher compared to the moderate-to-high transmission area. Much higher health policy maker WTPs were required to deem mRDT introduction a cost-effective intervention in the low transmission area (Figures 4b and 5b). From a health sector perspective, a policy maker WTP of US$13.5 per appropriately treated child would lead to a probability of 83% that CHWs using mRDTs to diagnose malaria was a cost-effective intervention; whilst from a societal perspective, the probability of mRDTs performed by CHWs being cost-effective in the low transmission area was 87% if the WTP was US$20.0.

## Discussion

This analysis presents a comprehensive assessment of the economic effects of introducing mRDT-based diagnosis into community-based treatment services provided by CHWs, including data on the health sector and household costs arising from reduced use of ACTs and clinical management of mRDT negative patients, in addition to implementation costs. Use of data derived from randomised trials, permits measurement of the extra (incremental) cost and effects if mRDTs were supplied to CHWs who previously prescribed ACT treatment to children under five based on presumptive diagnosis; whilst a common methodology across the two trials generates comparable data on costs and cost-effectiveness for two contrasting transmission settings within the same health system. In the moderate-to-high transmission setting, the incremental cost per additional child appropriately treated by CHWs using mRDTs and ACTs was low; at a total cost of US$3.0 from a health sector perspective and US$3.6 from a broader societal perspective For comparison, this is substantially lower than the range of health sector cost of US$5.0 to US$18.6 per appropriately treated patient of introducing mRDTs in public health centres found in different African countries (Batwala *et al.* 2011; Ansah *et al.* 2013; Mangham-Jefferies *et al.* 2014; Tawiah *et al.* 2016). An important explanation for the very low ICER of allowing trained CHWs to utilise mRDTs was the substantial improvement in the appropriate targeting of ACT treatment from 31% in the presumptive arm to 79% in the mRDT arm (48% points) seen in this trial. As a result of these low incremental cost-effectiveness ratios, the introduction of mRDT diagnosis is likely to be considered cost-effective, but as pointed out by Marseille *et al.* (2015) it is up to government policy makers in individual countries to decide the cost-effectiveness threshold below which the intervention will be regarded as cost-effective.

In the low transmission area, an even larger improvement in appropriate treatment for malaria from 8% to 90% (82% points) followed the introduction of mRDTs. However, due to higher absolute health sector costs in both arms and a large difference in costs between arms, the ICERs were much higher than in the moderate-to-high transmission area. These cost characteristics could be explained by substantially lower attendance in the low transmission area in particular the mRDT arm. While not formally investigated, possible explanations for the low attendance rate might include the following: low incidence of fever due to low malaria transmission, the presence of an alternative health provider in the form of a mission hospital and successful penetration during community sensitisation of the message that ‘not all fevers are malaria’ possibly resulting in patients being less likely to seek treatment from ‘malaria-only’ service providers like CHWs in a low transmission area. This finding reveals that CHWs are not a low cost service in all situations, confirming previous reports from a multi-country study incorporating data from seven countries in sub-Saharan Africa where areas with low utilisation CHW services combined with significant fixed costs led to high cost per service (Collins *et al.* 2014). Nonetheless, there may be ways of limiting the cost of providing services in low transmission areas. Illustrative cost calculations under the assumption that one CHW per village would be sufficient resulted in considerably lower ICERs of US$5.4 and US$6.9 from health sector and societal perspectives. Health policy makers may therefore wish to adjust the number of CHWs according to known transmission levels when planning community-based treatment programmes incorporating mRDT diagnosis. If CHWs are trained to treat multiple common infections, such as for integrated community case management (iCCM), this may lead to an overall increase in attendance, even in areas of low malaria transmission and subsequently result in a lower unit cost per patient treated.

Household cost constituted an important component of societal costs in both transmission settings. The responses of caregivers during interviews with respect to their out-of-pocket expenditure for treatment-seeking, purchasing special food to improve the health of the ill child and time unable to perform usual activities varied substantially, resulting in wide confidence intervals for incremental societal cost in both transmission settings. As a consequence, the uncertainty facing the health policy maker in the decision to allow CHWs to utilise mRDTs was much greater from a societal perspective compared to a more narrow health sector perspective. For example, in the moderate-to-high transmission area, to reach a 95% probability of mRDT use being cost-effective from a societal perspective would require a WTP of US$18.0 for each additional appropriately treated child; whereas a low WTP of US$3.5 would be sufficient to deem mRDTs cost-effective with almost 100% probability from a health sector perspective.

One of the frequently stated advantages of CHWs is that they increase access to malaria treatment for community members and lower costs of treatment-seeking. In the moderate-to-high transmission area of this study, households indeed experienced low out-of-pocket expenditure for visiting CHWs, completing referral and additional treatment-seeking in the private sector compared to household costs where individuals sought treatment at public health centres in Ghana (Tawiah *et al.* 2016). However, the situation was markedly different in the low transmission area where households experienced much higher out-of-pocket expenditure especially in the mRDT arm where the probability of additional treatment-seeking in the private sector was 36% after visiting CHWs (versus 11% in the presumptive arm). Visiting CHWs using mRDTs in a low transmission area, caregivers will more frequently be informed that the child does not suffer from malaria thus leading to additional treatment-seeking to treat the fever. Although the provision of a broader service package like iCCM in which CHWs are also trained to treat pneumonia and diarrhoea may diminish the number of febrile children that cannot be treated at community level, it may not fully obviate the need for additional treatment-seeking and resultant cost, particularly in low malaria transmission settings.

## Limitations

Data on household costs over a 14-day period were collected retrospectively through interviews with caregivers. Responses to questions regarding out-of-pocket expenditure and time utilisation varied a lot in the sample resulting in standard deviations more than double the mean value for many of the household cost categories. This may reflect the methodological limitations inherent in this retrospective approach, such as accuracy of recall, but may also reflect genuine variation in household behaviour when facing a sick child with fever. The variation was larger than assumed in the sample size calculation for the household cost interviews, and a larger sample of household interviews would therefore have been desirable to reduce uncertainty of the ICERs from a societal perspective in the form of narrower confidence intervals.

## Conclusions

Availability of mRDTs among CHWs resulted in a substantial improvement in appropriate treatment of children compared to a situation with presumptive diagnosis in both transmission settings. In the moderate-to-high transmission area this was achieved at low cost, resulting in a low ICER from both health sector and societal perspectives. In contrast, much higher health sector cost and ICERs were found in the low transmission area reflecting the necessity that significant resources must be invested in a programme before CHWs can start offering their services, combined with low attendance for malaria treatment. Costs could be substantially reduced if the number of CHWs per community who were focused on providing malaria care was lower in areas with low attendance thus saving some of the fixed costs related to individual CHWs such as training and equipment.

## Supplementary Material

Supplementary Data

## References

[czw171-B1] Ajayi IO, Browne EN, Bateganya F 2008 Effectiveness of artemisinin-based combination therapy used in the context of home management of malaria: a report from three study sites in sub-Saharan Africa. Malaria Journal 7: 190. 18822170 10.1186/1475-2875-7-190 PMC2567328

[czw171-B2] Ansah EK, Epokor M, Whitty CJ 2013 Cost-effectiveness analysis of introducing RDTs for malaria diagnosis as compared to microscopy and presumptive diagnosis in central and peripheral public health facilities in Ghana. American Journal of Tropical Medicine and Hygiene 89: 724–36. 23980131 10.4269/ajtmh.13-0033 PMC3795104

[czw171-B3] Baiden F, Bruce J, Webster J 2016 Effect of test-based versus presumptive treatment of malaria in under-five children in rural Ghana—a cluster-randomised trial. PLoS One 11: e0152960. 27055275 10.1371/journal.pone.0152960 PMC4824463

[czw171-B4] Batwala V, Magnussen P, Hansen KS 2011 Cost-effectiveness of malaria microscopy and rapid diagnostic tests versus presumptive diagnosis: implications for malaria control in Uganda. Malaria Journal 10: 372. 22182735 10.1186/1475-2875-10-372 PMC3266346

[czw171-B5] Black RE, Cousens S, Johnson HL 2010 Global, regional, and national causes of child mortality in 2008: a systematic analysis. Lancet 375: 1969–87. 20466419 10.1016/S0140-6736(10)60549-1

[czw171-B6] Briggs A, Claxton C, Sculpher M. 2006 Decision Modelling for Health Economic Evaluation. Oxford: Oxford University Press.

[czw171-B7] Brouwer W, Rutten F, Koopmanschap M. 2001 Costing in economic evaluations In: Drummond M, McGuire A (eds). Economic Evaluation in Health Care. Oxford: Oxford University Press.

[czw171-B8] Chanda P, Hamainza B, Moonga HB 2011a Community case management of malaria using ACT and RDT in two districts in Zambia: achieving high adherence to test results using community health workers. Malaria Journal 10: 158. 21651827 10.1186/1475-2875-10-158 PMC3121653

[czw171-B9] Chanda P, Hamainza B, Moonga HB 2011b Relative costs and effectiveness of treating uncomplicated malaria in two rural districts in Zambia: implications for nationwide scale-up of home-based management. Malaria Journal 10: 159. 21651828 10.1186/1475-2875-10-159 PMC3121654

[czw171-B10] Chinbuah AM, Gyapong JO, Pagnoni F 2006 Feasibility and acceptability of the use of artemether-lumefantrine in the home management of uncomplicated malaria in children 6-59 months old in Ghana. Tropical Medicine and International Health 11: 1003–16. 16827701 10.1111/j.1365-3156.2006.01654.x

[czw171-B11] Collins D, Jarrah Z, Gilmartin C 2014 The costs of integrated community case management (iCCM) programs: a multi-country analysis. Journal of Global Health 4: 020407. 25520797 10.7189/jogh.04.020407 PMC4267093

[czw171-B12] Conteh L, Walker D. 2004 Cost and unit cost calculations using step-down accounting. Health Policy and Planning 19: 127–35. 14982891 10.1093/heapol/czh015

[czw171-B13] Counihan H, Harvey SA, Sekeseke-Chinyama M 2012 Community health workers use malaria rapid diagnostic tests (RDTs) safely and accurately: results of a longitudinal study in Zambia. Journal of Tropical Medicine and Hygiene 87: 57–63. 10.4269/ajtmh.2012.11-0800 PMC3391058 22764292

[czw171-B14] Darmstadt GL, Bhutta ZA, Cousens S 2005 Evidence-based, cost-effective interventions: how many newborn babies can we save? Lancet 365: 977–88. 15767001 10.1016/S0140-6736(05)71088-6

[czw171-B100] Doubilet P, Begg CB, Weinstein MC *et al* 1984. Probabilistic sensitivity analysis using Monte Carlo simulation. A practical approach. *Medical Decision Making* **5**: 157–77. 10.1177/0272989X8500500205 3831638

[czw171-B15] Drummond MF, Sculpher MJ, Torrance GW 2005 Methods for the Economic Evaluation of Health Care Programmes. Oxford: Oxford University Press.

[czw171-B16] Elmardi KA, Malik EM, Abdelgadir T 2009 Feasibility and acceptability of home-based management of malaria strategy adapted to Sudan's conditions using artemisinin-based combination therapy and rapid diagnostic test. Malaria Journal 8: 39. 19272157 10.1186/1475-2875-8-39 PMC2660358

[czw171-B17] Fenwick E, O'Brien BJ, Briggs A. 2004 Cost‐effectiveness acceptability curves–facts, fallacies and frequently asked questions. Health Economics 13: 405–15. 15127421 10.1002/hec.903

[czw171-B18] Gaumer G, Zeng W, Nandakumar AK. 2014 Modelling the returns on options for improving malaria case management in Ethiopia. Health Policy and Planning 29: 998–1007. 24197404 10.1093/heapol/czt081

[czw171-B19] Haines A, Sanders D, Lehmann U 2007 Achieving child survival goals: potential contribution of community health workers. Lancet 369: 2121–31. 17586307 10.1016/S0140-6736(07)60325-0

[czw171-B20] Harvey SA, Jennings L, Chinyama M 2008 Improving community health worker use of malaria rapid diagnostic tests in Zambia: package instructions, job aid and job aid-plus-training. Malaria Journal 7: 160. 18718028 10.1186/1475-2875-7-160 PMC2547110

[czw171-B21] Hawkes M, Katsuva JP, Masumbuko CK. 2009 Use and limitations of malaria rapid diagnostic testing by community health workers in war-torn Democratic Republic of Congo. Malaria Journal 8: 308. 20028563 10.1186/1475-2875-8-308 PMC2804690

[czw171-B22] Hayes RJ, Bennett S. 1999 Simple sample size calculation for cluster-randomized trials. International Journal of Epidemiology 28: 319–26. 10342698 10.1093/ije/28.2.319

[czw171-B23] Jones G, Steketee RW, Black RE 2003 How many child deaths can we prevent this year? Lancet 362: 65–71. 12853204 10.1016/S0140-6736(03)13811-1

[czw171-B24] Kamal-Yanni MM, Potet J, Saunders PM. 2012 Scaling-up malaria treatment: a review of the performance of different providers. Malaria Journal 11: 414. 23231707 10.1186/1475-2875-11-414 PMC3547718

[czw171-B25] Lal S, Ndyomugenyi R, Alexander ND 2015 Health facility utilisation changes during the introduction of community case management of malaria in South Western Uganda: an interrupted time series approach. PLoS One 10: e0137448. 26356099 10.1371/journal.pone.0137448 PMC4565684

[czw171-B26] Leslie T, Mikhail A, Mayan I 2014 Rapid diagnostic tests to improve treatment of malaria and other febrile illnesses: patient randomised effectiveness trial in primary care clinics in Afghanistan. BMJ 348: g3730. 24948695 10.1136/bmj.g3730 PMC4064827

[czw171-B27] Liu L, Johnson HL, Cousens S 2012 Global, regional, and national causes of child mortality: an updated systematic analysis for 2010 with time trends since 2000. Lancet 379: 2151–61. 22579125 10.1016/S0140-6736(12)60560-1

[czw171-B28] Löthgren M., Zethraeus N. 2000 Definition, interpretation and calculation of cost‐effectiveness acceptability curves. Health Economics 9: 623–30. 11103928 10.1002/1099-1050(200010)9:7<623::aid-hec539>3.0.co;2-v

[czw171-B29] Lubell Y, Mills AJ, Whitty CJ 2010 An economic evaluation of home management of malaria in Uganda: an interactive Markov model. PLoS One 5: e12439. 20805977 10.1371/journal.pone.0012439 PMC2929190

[czw171-B30] Management Sciences for Health. 2012. International Drug Price Indicator Guide 2011. Medford, Massachusetts: Management Sciences for Health.

[czw171-B31] Mangham-Jefferies L, Wiseman V, Achonduh OA 2014 Economic evaluation of a cluster randomized trial of interventions to improve health workers' practice in diagnosing and treating uncomplicated malaria in Cameroon. Value in Health 17: 783–91. 25498773 10.1016/j.jval.2014.07.010

[czw171-B32] Marseille E, Larson B, Kazi DS 2015 Thresholds for the cost-effectiveness of interventions: alternative approaches. Bulletin of the World Health Organization 93: 118–24. 25883405 10.2471/BLT.14.138206 PMC4339959

[czw171-B101] Mbonye AK, Magnussen P, Lal S *et al* 2015. A cluster randomised trial introducing rapid diagnostic tests into the private health sector in Uganda: Impact on appropriate treatment of malaria. *PLoS One* **10**: e0129545. 10.1371/journal.pone.0129545 PMC4511673 26200467

[czw171-B33] Ministry of Health. 2010 Uganda Clinical Guidelines 2010: National Guidelines on Management of Common Conditions. Kampala, Uganda: Ministry of Health.

[czw171-B34] Mubi M, Janson A, Warsame M 2011 Malaria rapid testing by community health workers is effective and safe for targeting malaria treatment: randomised cross-over trial in Tanzania. PLoS One 6: e19753. 21750697 10.1371/journal.pone.0019753 PMC3130036

[czw171-B35] Ndyomugyenyi R, Magnussen P, Lal S 2016 Impact of malaria rapid diagnostic tests by community health workers on appropriate targeting of artemisinin-based combination therapy: randomized trials in two contrasting areas of high and low malaria transmission in south western Uganda. Tropical Medicine and International Health 21: 1157–70. 27383558 10.1111/tmi.12748 PMC5031222

[czw171-B36] Nonvignon J, Chinbuah MA, Gyapong M 2012 Is home management of fevers a cost-effective way of reducing under-five mortality in Africa? The case of a rural Ghanaian District. Tropical Medicine and International Health 17: 951–7. 22643324 10.1111/j.1365-3156.2012.03018.x

[czw171-B37] O’Brien BJ, Briggs AH. 2002 Analysis of uncertainty in health care cost-effectiveness studies: an introduction to statistical issues and methods. Statistical Methods in Medical Research 11: 455–68. 12516984 10.1191/0962280202sm304ra

[czw171-B38] Okwundu CI, Nagpal S, Musekiwa A 2013 Home- or community-based programmes for treating malaria. Cochrane Database of Systematic Reviews 5: CD009527. 10.1002/14651858.CD009527.pub2 PMC6532579 23728693

[czw171-B39] Perry HB, Zulliger R, Rogers MM. 2014 Community health workers in low-, middle-, and high-income countries: an overview of their history, recent evolution, and current effectiveness. Annual Review of Public Health 35: 399–421. 10.1146/annurev-publhealth-032013-182354 24387091

[czw171-B40] Ruizendaal E, Dierickx S, Peeters Grietens K 2014 Success or failure of critical steps in community case management of malaria with rapid diagnostic tests: a systematic review. Malaria Journal 13: 229. 24924295 10.1186/1475-2875-13-229 PMC4084582

[czw171-B41] Shillcutt SD, Walker DG, Goodman CA 2009 Cost effectiveness in low- and middle-income countries: a review of the debates surrounding decision rules. Pharmacoeconomics 27: 903–17. 19888791 10.2165/10899580-000000000-00000 PMC2810517

[czw171-B42] Sirima SB, Konaté A, Tiono AB 2003 Early treatment of childhood fevers with pre-packaged antimalarial drugs in the home reduces severe malaria morbidity in Burkina Faso. Tropical Medicine and International Health 8: 133–9. 12581438 10.1046/j.1365-3156.2003.00997.x

[czw171-B43] Staedke SG, Mwebaza N, Kamya MR 2009 Home management of malaria with artemether-lumefantrine compared with standard care in urban Ugandan children: a randomised controlled trial. Lancet 373: 1623–31. 19362361 10.1016/S0140-6736(09)60328-7

[czw171-B44] Tawiah T, Hansen KS, Baiden F 2016 Cost-effectiveness analysis of test-based versus presumptive treatment of malaria in children under five years in Central Ghana. PLoS One 11: e0164055. 27695130 10.1371/journal.pone.0164055 PMC5047443

[czw171-B45] Thiam S, Thwing J, Diallo I 2012 Scale-up of home-based management of malaria based on rapid diagnostic tests and artemisinin-based combination therapy in a resource-poor country: results in Senegal. Malaria Journal 11: 334. 23009244 10.1186/1475-2875-11-334 PMC3507725

[czw171-B46] Tiono AB, Kaboré Y, Traoré A 2008 Implementation of Home based management of malaria in children reduces the work load for peripheral health facilities in a rural district of Burkina Faso. Malaria Journal 7: 201. 18834504 10.1186/1475-2875-7-201 PMC2570683

[czw171-B47] Vaughan K, Kok MC, Witter S 2015 Costs and cost-effectiveness of community health workers: evidence from a literature review. Human Resources for Health 13: 71. 26329455 10.1186/s12960-015-0070-y PMC4557864

[czw171-B48] Walker D, Kumaranayake L. 2002 Allowing for differential timing in cost analyses: discounting and annualization. Health Policy and Planning 17: 112–8. 11861593 10.1093/heapol/17.1.112

[czw171-B49] Whitty CJ, Chandler C, Ansah E 2008 Deployment of ACT antimalarials for treatment of malaria: challenges and opportunities. Malaria Journal 7: S7. 19091041 10.1186/1475-2875-7-S1-S7 PMC2604871

[czw171-B50] WHO. 2006 Working Together for Health: The World Health Report 2006. Geneva, Switzerland: World Health Organization.

[czw171-B51] WHO. 2010 Guidelines for the Treatment of Malaria, 2nd edn Geneva, Switzerland: World Health Organization.

[czw171-B52] WHO. 2015 World Malaria Report 2015. Geneva, Switzerland: World Health Organization.

[czw171-B53] World Bank. 2015 World Bank Indicators. http://data.worldbank.org/indicator, accessed 30 October 2015.

[czw171-B54] Young M, Wolfheim C, Marsh DR 2012 World Health Organization/United Nations Children's Fund joint statement on integrated community case management: an equity-focused strategy to improve access to essential treatment services for children. American Journal of Tropical Medicine and Hygiene 87: 6–10. 23136272 10.4269/ajtmh.2012.12-0221 PMC3748523

