# Supplementary material for: Cost-effectiveness analysis of malaria rapid diagnostic tests for appropriate treatment of malaria at the community level in Uganda
Source: Health Policy Plan. 2017 Feb 15;32(5):676–89. doi: 10.1093/heapol/czw171 (PMC5406761; doi:10.1093/heapol/czw171)
Supplement: Supplementary Data [file czw171_Supp.zip › czw171-suppl_data/supplementary table 1b.docx]

**Supplementary Table 1b. Parameters utilised in decision model and distributions for probabilistic sensitivity analyses (PSA), incremental cost-effectiveness analysis of replacing presumptive diagnosis by rapid diagnostic tests performed by community health workers in a low transmission area in Rukungiri District, Uganda, 2011 (US$1=UGX2523).**

|  | ----------- Value ----------- | |  |  |
| --- | --- | --- | --- | --- |
| Parameter | mRDT  arm | Presumptive  arm | Source | Distribution  in PSA |
| Malaria positivity rate among children  under 5 visiting CHWs (%) | 6.0 | 5.6 | # | Point estimate |
| Sensitivity of diagnosis (%) | 20.8 | 91.3 | # | Beta |
| Specificity of diagnosis (%) | 98.2 | 2.9 | # | Beta |
| Adherence to positive mRDT result (%) | 75.0 | NA | # | Beta |
| Adherence to negative mRDT result (%) | 95.1 | NA | # | Beta |
| Community sensitisation, cost per visit (US$) | 0.9 | 0.5 | λ | Point estimate |
| Training of CHWs, cost per visit (US$) | 2.6 | 1.1 | λ | Point estimate |
| Supervision, cost per visit (US$) | 4.5 | 1.8 | λ | Point estimate |
| Allowances for CHWs, cost per visit (US$) | 10.3 | 5.5 | λ | Point estimate |
| Equipment for CHWs, cost per visit (US$) | 2.3 | 1.3 | λ | Point estimate |
| Supplies for CHWs, cost per visit (US$) | 1.2 | 0.7 | λ | Point estimate |
| Cost per mRDT, borne by the health sector (US$) | 1.0 | NA | β | Point estimate |
| Cost per ACT course for below 3 years, borne by  the health sector (US$) | 0.8 | 0.8 | β | Point estimate |
| Cost per ACT course for 3-7 years, borne by the  health sector (US$) | 1.6 | 1.6 | β | Point estimate |
| Cost per rectal artesunate, borne by the  health sector (US$) | 0.7 | 0.7 | β | Point estimate |
| CHW time for overhead activities,  value per visit (US$) | 0.5 | 0.2 | π | Gamma |
| CHW time diagnosis and treatment,  value per visit (US$) | 0.1 | 0.1 | π | Gamma |
| Probability of referral and seeking care at  public health centres (%) | 8.7 | 1.8 | # | Beta |
| Malaria diagnosis and treatment at health centre,  child below 3 years, cost per visit (US$) | 2.7 | 2.7 | μ | Point estimate |
| Malaria diagnosis and treatment at health centre,  child aged 3-7 years, cost per visit (US$) | 3.8 | 3.8 | μ | Point estimate |
| Treatment with antibiotics at health centre,  child below 1 year, cost per visit (US$) | 1.8 | 1.8 | μ | Point estimate |
| Treatment with antibiotics at health centre,  child aged 1-5 years, cost per visit (US$) | 1.9 | 1.9 | μ | Point estimate |
| Out-of-pocket expenditure per visit at CHWs (US$) | 0.2 | 0.0 | δ | Gamma |
| Out-of-pocket expenditure per visit to  public health centre (US$) | 0.6 | 0.6 | δ | Gamma |
| Probability of additional treatment-seeking  in the private sector (%) | 35.7 | 11.4 | δ | Beta |
| Out-of-pocket expenditure for drugs, fees and  transport per visit in the private sector (US$) | 3.8 | 3.8 | δ | Gamma |
| Out-of-pocket expenditure for special food  to improve health per child (US$) | 1.0 | 1.2 | δ | Gamma |
| Time utilised for caring for ill household  member per fever episode (days) | 1.4 | 1.3 | δ | Gamma |
| Value of lost time per day (US$) | 1.2 | 1.2 | € | Point estimate |

^#^ Ndyomugyenyi *et al*. (2016), ^λ^ Study accounting system for the trial, ^β^ Market price, adjusted (see text),

^π^ Interviews with CHWs, ^μ^ Average unit cost in four public health centres (see text), ^δ^ Household cost interviews (see text), ^€^ World Bank (2016).
