# Supplementary material for: Cost-effectiveness analysis of malaria rapid diagnostic tests for appropriate treatment of malaria at the community level in Uganda
Source: Health Policy Plan. 2017 Feb 15;32(5):676–89. doi: 10.1093/heapol/czw171 (PMC5406761; doi:10.1093/heapol/czw171)
Supplement: Supplementary Data [file czw171_Supp.zip › czw171-suppl_data/supplementary table 2.docx]

**Supplementary Table 2. Scenario analysis assuming one CHW per village.**

**Costs and effects in a standard population of 1000 children suspected of malaria by study arm and incremental cost-effectiveness ratio (ICER) of replacing presumptive diagnosis by rapid diagnostic tests performed by community health workers in a low transmission area in Rukungiri District, Uganda, 2011 (US$1=UGX2,523).**

|  | ------ mRDT arm ------ | | -- Presumptive arm -- | |
| --- | --- | --- | --- | --- |
|  |  |  |  |  |
|  | ***N*** | ***%*** | ***N*** | ***%*** |
| ***Children suspected of malaria*** | ***1,000*** | ***100*** | ***1,000*** | ***100*** |
| Malaria (according to reference diagnosis) | 60 | 6 | 56 | 6 |
| Treated with ACT | 69 | 7 | 968 | 97 |
| Appropriately treated ^#^ | 901 | 90 | 78 | 8 |
|  |  |  |  |  |
|  | ***US$*** | ***%*** | ***US$*** | ***%*** |
| ***Health sector cost per 1000 children*** | ***9,776*** | ***68*** | ***5,308*** | ***61*** |
| Community sensitisation | 899 | 6 | 462 | 5 |
| Training ^β^ | 871 | 6 | 384 | 4 |
| Supervision | 2,044 | 14 | 772 | 9 |
| Allowances for CHWs | 3,445 | 24 | 1,769 | 20 |
| Equipment for CHWs | 777 | 5 | 399 | 5 |
| mRDTs supplied | 933 | 6 | 0 | 0 |
| ACTs prescribed | 72 | 0 | 1,078 | 12 |
| Other supplies | 560 | 4 | 411 | 5 |
| Completed referrals to public health centres | 175 | 1 | 33 | 0 |
| ***CHW cost (value of time) per 1000 children*** | ***273*** | ***2*** | ***175*** | ***2*** |
| Overhead activities ^α^ | 148 | 1 | 72 | 1 |
| Diagnosis and treatment | 125 | 1 | 103 | 1 |
| ***Household cost per 1000 children*** | ***4,374*** | ***30*** | ***3,262*** | ***37*** |
| CHW visit (initial visit) | 164 | 1 | 14 | 0 |
| Completed referrals to public health centres | 49 | 0 | 9 | 0 |
| Drugs, fees, travel (private sector visits) | 1,371 | 10 | 439 | 5 |
| Special food to improve health | 960 | 7 | 1,200 | 14 |
| Opportunity cost of time lost | 1,829 | 13 | 1,600 | 18 |
| ***Total societal costs per 1000 children*** | ***14,424*** | ***100*** | ***8,745*** | ***100*** |
|  |  |  |  |  |
| **Incremental analysis** |  |  |  |  |
| **(Replace presumptive diagnosis by mRDT diagnosis** | |  |  |  |
| **in 1000 children suspected of malaria)** | |  |  |  |
| Incremental number of appropriately treated [95% CI] | | 822 | [794; 848] |  |
| Incremental health sector cost, US$ [95% CI] |  | 4,468 | [4,423; 4,518] | |
| Incremental societal cost, US$ [95% CI] |  | 5,679 | [-3,604; 14,797] | |
| ICER health sector perspective, US$ [95% CI] |  | 5.4 | [5.2; 5.7] |  |
| ICER societal perspective, US$ [95% CI] |  | 6.9 | [-4.4; 18.0] |  |

^#^ Child with a positive reference diagnosis prescribed an ACT or child with a negative reference diagnosis not prescribed an ACT.

^β^ Including cost of initial period of close support supervision.

^α^ Quarterly review meetings, collection of supplies, communication with supervisors, etc.
